# Simulated patient and role play methodologies for communication skills and empathy training of undergraduate medical students

**DOI:** 10.1186/s12909-020-02401-0

**Published:** 2020-12-04

**Authors:** Cristina Bagacean, Ianis Cousin, Anne-Helene Ubertini, Mohamed El Yacoubi El Idrissi, Anne Bordron, Lolita Mercadie, Leonor Canales Garcia, Jean-Christophe Ianotto, Philine De Vries, Christian Berthou

**Affiliations:** 1 Univ Brest, Inserm, UMR1227, Lymphocytes B et Autoimmunité, Brest, France; 2 Department of Clinical Hematology, CHU de Brest, Brest University Medical School Hospital, 2 Av Foch, 29609 Brest, France; 3 Department of Pediatric Surgery, CHU de Brest, Brest, France; 4 Univ Brest, Department of Humanities and Social Sciences (SHS), Brest, France; 5 Univ Brest, CeSIM, Brest, France

**Keywords:** Communication, Verbal, Non-verbal, Empathy, Simulated patient, Role play

## Abstract

**Background:**

Verbal and non-verbal communication, as well as empathy are central to patient-doctor interactions and have been associated with patients’ satisfaction. Non-verbal communication tends to override verbal messages. The aim of this study was to analyze how medical students use verbal and non-verbal communication using two different educational approaches, student role play (SRP) and actor simulated patient (ASP), and whether the non-verbal behaviour is different in the two different poses.

**Methods:**

Three raters evaluated 20 students playing the doctor role, 10 in the SRP group and 10 in the ASP group. The videos were analyzed with the Calgary-Cambridge Referenced Observation Guide (CCG) and, for a more accurate evaluation of non-verbal communication, we also evaluated signs of nervousness, and posture. Empathy was rated with the CARE questionnaire. Independent Mann Whitney U tests and Qhi square tests were performed for statistical analysis.

**Results:**

From the 6 main tasks of the CCG score, we obtained higher scores in the ASP group for the task ‘Gathering information’ (*p* = 0.0008). Concerning the 17 descriptors of the CCG, the ASP group obtained significantly better scores for ‘Exploration of the patients’ problems to discover the biomedical perspective’ (*p* = 0.007), ‘Exploration of the patients’ problems to discover background information and context’ (*p* = 0.0004) and for ‘Closing the session – Forward planning’ (*p* = 0.02). With respect to non-verbal behaviour items, nervousness was significantly higher in the ASP group compared to the SRP group (*p* < 0.0001). Concerning empathy, no differences were found between the SRP and ASP groups.

**Conclusions:**

Medical students displayed differentiated verbal and non-verbal communication behaviour during the two communication skills training methodologies. These results show that both methodologies have certain advantages and that more explicit non-verbal communication training might be necessary in order to raise students’ awareness for this type of communication and increase doctor-patient interaction effectiveness.

## Background

Effective communication and empathy are central to patient-doctor interactions and have been shown to be associated with patients’ satisfaction, disclosure, compliance, and ultimately, with the outcomes of the medical process [1–3]. Moreover, patient-centered care was associated with decreased utilization of health care services and lower total annual charges [4]. Therefore, programs on communication skills training, including empathy as the ability to communicate an understanding of a patient’s world and to act on that understanding in a therapeutic way, have a well established value in the medical curricula [4, 5]. Communication in medical encounters comprises verbal and non-verbal aspects. Non-verbal messages tend to override the verbal messages and as reported by Mehrabian and Ferris, the impact of the verbal communication represents only 7% from the total impact of communication [6, 7]. On the other side, vocal communication has an impact of 38% and facial communication, 55% [6]. Therefore, non-verbal signs of the physician like eye contact, posture, tone of voice, greatly influences patient’s disclosure and satisfaction [7, 8]. Empathy is also a critical point of the communication process and is associated with patients’ satisfaction and outcomes [9–11].

Based on the premise that communication skills and empathy are malleable qualities that can be learned and improved, educators are continuously improving their didactic methods in order to teach effectively medical communication and empathy [12–14]. Interactive approaches that typically rely on role play, simulated or standardized patients are now favored [15, 16].

Taking into account that health systems worldwide are facing shortages in terms of resources, including their resources for medical education, the student role play (SRP) represents a cost-free, and therefore, an attractive approach for medical curriculum designers [17]. Previous research has shown that there is a particular educational benefit to SRP, regarding students’ understanding of the patient perspective, which can be achieved by playing not only the doctor role, but also the role of the patient [18]. Concerning the professional actor simulated patient (ASP) approach, it represents an expensive tool for communication skills training but students and faculty value mostly the realism and the depth of emotion of the situation, and the layperson perspective and feedback of the actor [19, 20].

There are several studies demonstrating comparable performance levels between ASP and SRP, but there is a cost-effectiveness advantage for SRP [17, 18]. However, there are few studies that evaluate the most impactful part of the communication process, the non-verbal communication [6]. In this context, the aim of our study was to analyze whether and how well medical students from the Faculty of Medicine of Brest use skills of verbal and non-verbal communication in the 2 different approaches, SRP and ASP. We also wanted to identify if the well recognized higher authenticity of the ASP approach generates different non-verbal behaviors as compared to the SRP approach. The non-verbal behaviors are generally under less conscious control but if recognized, this type of communication can also be auto-controlled and improved [21].

## Methods

### Educational workshops and participants

Since 2016, the undergraduate curricula at the Faculty of Medicine of Brest, France, includes a communication skills training program, with a particular focus on empathy. Researchers identified two types of empathy: affective and cognitive. Affective empathy refers to sensations and feelings in response to another person’s emotions, while cognitive empathy refers to the ability to identify and understand emotions of others [22]. During the communication skills training program, we are helping students to identify and understand situations, feelings, motives, and perspectives, and, moreover, recognize and appreciate concerns of another person; we are trying to cultivate medical students’ curiosity about patients and challenge prejudice [23].

The program is relying on an interactive approach by using SRP for the fourth year medical students and ASP for the fifth year students. The communication skills training program is realised at the Simulation Center (CeSIM) of the Faculty of Medicine.

Communication skills training workshops of the forth year students were facilitated by a general physician and a communication specialist. The duration of the workshops was of 6 h, during which, 15 students were portraying the role of patients, family members and doctors. The enacted cases were based on typical or usual presentations of asthma or coronary disease in a patient who is denying and minimising the disease and who is accompanied in consultation by a worried family member.

The fifth year medical students communication skills training workshops were facilitated by a clinical haematologist, a clinical psychologist and 2 professional actors. The duration of the workshops was of 4 h and 15 learners were participating. During the workshop, 2 medical students portraying the doctors interacted directly with 2 professional actors portraying the patient and a family member. Actors are trained to portray a patient in a clinical scenario, they are not given a specific script. Instead, they receive all the details of the case including symptoms, signs, medical history, patient’s education and socioeconomic status. Based on this information, they improvise their encounters with the medical students. The simulated patients are encouraged to go with their own emotional response to the situation. The 2 enacted cases represented 2 different times of the same clinical situation based on a lymphoid disease presentation. The first time the ASP presented systemic symptoms such as weight loss, night sweats and fever as well as an inflammatory low back pain. The second time, the doctor saw the patient with the results of the exams realised and had to explain to the ASP the presence of a retroperitoneal adenopathy observed on the CT scan that needs further exploration by biopsy. The ASP was initially denying and minimising the symptoms, but became more aware of the situation in the second scenario. For the 2 consultation times, the ASP was accompanied in consultation by a second actor playing the role of a worried family member.

Learners not directly implicated in the SRP and ASP situations observed the interaction through a closed circuit video, in another room. All medical simulation interviews were filmed and recorded. Each enactment was immediately followed by a formal debriefing led by facilitators and including all the learners.

### Sample studied, student participants and raters

Ten SRP videos and 10 ASP videos were randomly selected from our data base. The standards of the Declaration of Helsinki were maintained and all participants to the study gave their written consent. The videos were then analyzed by 3 raters, who carried out data collection independently. Raters were represented by a clinical haematologist, a paediatric surgeon and an expert in communication.

Over the 2 sessions, 20 students participated in the study, 9 (45%) male and 11 (55%) female (4 male in the SRP group and 5 in the ASP group, 6 female in the SRP group and 5 in the ASP group).

### Evaluation criteria

For the observation of the videos, 4 different instruments have been used. A global assessment of the communication skills was done by using the short version of the Calgary-Cambridge Referenced Observation Guide (CCG) (Supplementary Table 1) [13, 24, 25]. The CCG assessment grids contained 6 tasks with 17 descriptors, which had to be rated on a 3 point Likert scale (1: Not done; 2: Incompletely done; 3: Done). Although the CCG assesses the non-verbal behavior and empathy, in order to have a more accurate evaluation of these items, we divided the non-verbal behavior criterion into two parts: signs of nervousness, rated on a 4 point Likert scale (1: None; 2: Low; 3: Medium; 4: High), and posture (appropriate/inappropriate) during the first and the last 5 min of the session, as well as the global posture. Empathy was rated with the CARE (Consultation and Relational Empathy) questionnaire which contains 10 items (Supplementary Table 2) [26–29]. The CARE scoring system was developed initially for assessment of physicians’ empathy by patients, but we used it in our study for external rating of empathy. Each item was rated on a 5-point Likert scale (1, Poor; 2: Fair; 3: Good; 4: Very good, and 5: Excellent). All ten items were then added, giving a maximum possible score of 50, and a minimum of 10.

### Statistical analysis

Independent Mann Whitney U tests were performed to identify whether the two groups SRP and ASP yielded different CCG scores, CARE scores and nervousness scores. Qhi square tests were performed for posture evaluations. The same statistical tests were performed to identify gender communication skills differences. *P* values under 0.05 were considered significant. Statistical analyses were performed using GraphPad Prism 7.0 (La Jolla, CA). Effect size d and Power (1-β err prob) were calculated with G Power 3.1.9.7. An effect size *d* ≤ 0.2 was considered a ‘small’ effect size,0.2 ≤ d ≤ 0.5 represents a ‘medium’ effect size and d ≥ 0.8 a ‘large’ effect size. Reliability of agreement between the 3 raters was assesed by calculating Fleiss Kappa with the ReCal3 0.1 software (http://dfreelon.org/recal/recal3.php).

## Results

### Communication skills differences between SRP and ASP groups

Independent t-tests were performed in order to identify if the 2 groups SRP (*n* = 10) and ASP (*n =* 10) yielded different CCG scores (Table 1). The overall CCG scores difference between the 2 groups did not reach statistical significance. The reliability of agreement between the 3 raters concerning the CCG scores was substantial, with a Fleiss kappa (FK) = 0.72 for the SRP group and FK = 0.74 for the ASP group [32]. From the 6 main tasks, namely initiating the session, gathering information, explanation and planning, building the relationship, providing structure and closing the session, we obtained a significant statistical difference for the task ‘gathering information’, with higher scores attained by the ASP group (SRP group mean = 7.36 ± 1.45, ASP group mean = 8.46 ± 0.73, *p* = 0.0008, effect size d = 0.95, Power (1-β err prob) = 0.53). Concerning the 17 descriptors of the CCG, we obtained higher scores in the ASP group for ‘Gathering information – Exploration of the patients’ problems to discover the biomedical perspective’ (SRP group mean = 2.36 ± 0.76, ASP group mean = 2.83 ± 0.37, *p* = 0.007, effect size d = 0.79, Power (1-β err prob) = 0.38), ‘Gathering information – Exploration of the patients’ problems to discover background information and context’ (SRP group mean = 2.26 ± 0.69, ASP group mean = 2.83 ± 0.37, *p* = 0.0004, effect size d = 1.02, Power (1-β err prob) = 0.59) and for ‘Closing the session – Forward planning’ (SRP group mean = 2.46 ± 0.68, ASP group mean = 2.80 ± 0.48, *p* = 0.02, effect size d = 0.58, Power (1-β err prob) = 0.23).

**Table 1:** Calgary-Cambridge guide scores for the student role play and actor simulated patient groups. Adapted from Kurtz SM, 1998 and Silverman JD, 1998 [30, 31]

CCG main tasks and descriptors	SRP group (*n* = 10) Mean ± SD	ASP group (*n* = 10) Mean ± SD	*p* (Mann Whitney U test)	Effect size d	Power (1-β err prob)
**Initiating the session**	7.80 ± 1.23	8.23 ± 0.97	0.16	0.38	0.12
Preparing the session	2.13 ± 0.81	2.43 ± 0.72	0.14	0.39	0.13
Establishing initial rapport	2.83 ± 0.37	2.86 ± 0.34	0.72	0.08	0.05
Identifying the reasons for the consultation	2.83 ± 0.46	2.93 ± 0.25	0.38	0.27	0.09
**Gathering information - exploration of the patient’s problems to discover the:**	7.36 ± 1.45	8.46 ± 0.73	0.0008	0.95	0.53
biomedical perspective	2.36 ± 0.76	2.83 ± 0.37	0.007	0.79	0.38
the patient’s perspective	2.73 ± 0.44	2.80 ± 0.40	0.55	0.17	0.06
background information - context	2.26 ± 0.69	2.83 ± 0.37	0.0004	1.02	0.59
**Explanation and planning**	10.67 ± 1.56	10.07 ± 2.22	0.43	0.31	0.1
Providing the correct amount and type of information	2.53 ± 0.57	2.30 ± 0.70	0.2	0.36	0.12
Aiding accurate recall and understanding	2.76 ± 0.50	2.46 ± 0.68	0.05	0.50	0.19
Achievieng a shared understanding: incorporating the patient’s illness framework	2.73 ± 0.52	2.60 ± 0.62	0.38	0.23	0.19
Planning: shared decision making	2.63 ± 0.61	2.70 ± 0.53	0.73	0.12	0.06
**Building the relationship**	8.10 ± 1.15	7.66 ± 1.18	0.11	0.37	0.12
Using appropriate non-verbal behaviour	2.73 ± 0.52	2.53 ± 0.62	0.17	0.34	0.11
Developping rapport	2.7 ± 0.53	2.53 ± 0.50	0.14	0.33	0.11
Involving the patient	2.66 ± 0.60	2.60 ± 0.56	0.49	0.10	0.05
**Providing structure**	5.33 ± 1.09	5.63 ± 0.66	0.28	0.33	0.11
Making organisation overt	2.66 ± 0.60	2.76 ± 0.50	0.52	0.18	0.06
Attending to flow	2.66 ± 0.60	2.86 ± 0.34	0.17	0.41	0.14
**Closing the session**	5.13 ± 1.19	5.40 ± 1.07	0.26	0.23	0.08
Forward planning	2.46 ± 0.68	2.80 ± 0.48	0.02	0.58	0.23
Ensuring appropriate point of closure	2.66 ± 0.66	2.60 ± 0.72	0.74	0.37	0.12

Abbreviations: *CCG* Calgary-Cambridge guide, *SRP* student role play, *ASP* actor simulated patient, *SD* standard deviation

With respect to non-verbal behaviour items, nervousness was significantly higher in the ASP group compared to the SRP group (*p* < 0.0001, effect size d = 1.25, Power (1-β err prob) = 0.75, SRP group mean = 1.33 ± 0.54, ASP group mean = 2.36 ± 1.03). The reliability of agreement between the 3 raters concerning the nervousness was substantial, with a FK = 0.75 for the SRP group and FK = 0.72 for the ASP group. No significant difference was observed between the 2 groups for posture in the first 5 min, last five minutes and the entire session (SRP FK = 0.82, ASP FK = 0.65). The most frequent signs of nervousness observed by the raters were self-touching (rubbing one’s face, grooming the hair), frequent postural shift, manipulation of objects (mostly the pen) and inappropriate facial display such as smiling or laughing.

Concerning empathy, no differences were found between the SRP and ASP groups for the global CARE questionnaire or CARE items. The reliability of agreement between the 3 raters concerning CARE scores was substantial, with a FK = 0.72 for the SRP group and FK = 0.74 for the ASP group.

### Gender specific aspects of communication skills

As it is well known that communication styles of female physicians interacting with patients are consistently different from their male counterparts, we also compared the male and female students participating in the study [33].

Concerning the CCG scores, no significant differences were achieved between Female (*n* = 11) and male (*n* = 9) students for the global score but female students performed significantly better for the main task ‘Building the relationship’ (female group mean = 8.27 ± 1, male group mean = 7.40 ± 1.21, *p* = 0.002, effect size d = 0.78, Power (1-β err prob) = 0.38) and for the descriptors ‘Initiating the session – Identifying the reasons for the consultation’ (female group mean = 2.97 ± 0.17, male group mean = 2.77 ± 0.5, *p* = 0.04, effect size d = 0.53, Power (1-β err prob) = 0.20), and ‘Building the relationship – Using appropriate non-verbal behavior’ (female group mean = 2.8 ± 0.46, male group mean = 2.4 ± 0.63, *p* = 0.003, effect size d = 0.73, Power (1-β err prob) = 0.35) (Table 2). The FK concerning the CCG scores shows a substantial reliability of agreement between the 3 raters (Male FK = 0.72, Female FK = 0.72). With respect to non-verbal communication items, nervousness and posture and to empathy evaluated by the CARE score, no significant differences were found between the 2 gender groups.

**Table 2:** Calgary-Cambridge guide scores for male and female students. Adapted from Kurtz SM, 1998 and Silverman JD, 1998 [30, 31]

CCG main tasks and descriptors	Male (*n =* 9) Mean ± SD	Female (*n =* 11) Mean ± SD	*p* (Mann Whitney U test)	Effect size d	Power (1-β err prob)
**Initiating the session**	7.96 ± 1.34	8.06 ± 0.93	0.78	0.09	0.05
Preparing the session	2.37 ± 0.79	2.21 ± 0.78	0.39	0.20	0.07
Establishing initial rapport	2.81 ± 0.39	2.87 ± 0.33	0.5	0.17	0.06
Identifying the reasons for the consultation	2.77 ± 0.50	2.97 ± 0.17	0.04	0.53	0.20
**Gathering information - exploration of the patient’s problems to discover the:**	7.74 ± 1.25	8.06 ± 1.21	0.16	0.26	0.09
biomedical perspective	2.55 ± 0.75	2.63 ± 0.54	0.98	0.12	0.06
the patient’s perspective	2.70 ± 0.46	2.81 ± 0.39	0.3	0.25	0.08
background information - context	2.48 ± 0.57	2.60 ± 0.65	0.26	0.20	0.07
**Explanation and planning**	10.07 ± 2.2	10.61 ± 1.67	0.36	0.27	0.09
Providing the correct amount and type of information	2.37 ± 0.68	2.45 ± 0.61	0.68	0.12	0.06
Aiding accurate recall and understanding	2.51 ± 0.64	2.69 ± 0.58	0.2	0.29	0.09
Achievieng a shared understanding: incorporating the patient’s illness framework	2.55 ± 0.69	2.75 ± 0.43	0.32	0.35	0.11
Planning: shared decision making	2.63 ± 0.62	2.69 ± 0.52	0.77	0.10	0.06
**Building the relationship**	7.40 ± 1.21	8.27 ± 1.00	0.002	0.78	0.38
Using appropriate non-verbal behaviour	2.40 ± 0.63	2.81 ± 0.46	0.003	0.73	0.35
Developping rapport	2.48 ± 0.57	2.72 ± 0.45	0.08	0.46	0.17
Involving the patient	2.51 ± 0.70	2.72 ± 0.45	0.3	0.36	0.12
**Providing structure**	5.29 ± 1.17	5.63 ± 0.60	0.4	0.37	0.12
Making organisation overt	2.55 ± 0.69	2.84 ± 0.36	0.07	0.53	0.20
Attending to flow	2.74 ± 0.59	2.78 ± 0.41	0.92	0.08	0.05
**Closing the session**	5.29 ± 1.23	5.24 ± 1.06	0.56	0.04	0.05
Forward planning	2.63 ± 0.62	2.63 ± 0.60	0.99	0	0.05
Ensuring appropriate point of closure	2.66 ± 0.67	2.60 ± 0.70	0.68	0.08	0.05

Abbreviations: *CCG* Calgary-Cambridge guide, *SRP* student role play, *ASP* actor simulated patient, *SD* standard deviation

## Discussion

This study demonstrated that there is no significant difference between ASP and SRP in global communication skills evaluated by the CCG questionnaire. However, students’ communication skills were more appropriate when interacting with a professional actor rather than with a student colleague, in terms of gathering information in order to discover the biomedical perspective, background information and context but also in terms of closing the session with forward planning. When raters evaluated the items ‘gathering information’ in order to discover the biomedical perspective, background information and context, they evaluated if students: (1) tended to facilitate patient’s responses verbally or non-verbally, (2) paid more attention to the verbal and non-verbal cues (body language, facial expression, affect), (3) clarified patient’s statements that were unclear, (4) periodically summarized to verify own understanding of what the patient had said, (5) used concise, easily understood questions and comments, avoided or adequately explained jargon, (6) established dates and sequence of events. All the items before cited are linked to known valuable actor contributions like the higher authenticity and realism of the ASP situation as well as to the actors’ lack of medical knowledge, an important asset for the medical experience [19]. The actors’ flexibility in verbal and non-verbal communication as well as their emotional commitment seemed to generate more concentration/attention from the learner as well as a more adapted language. While colleagues know what to anticipate and have comparable clinical knowledge, actors are highly improvisational. There may be certain strategies and even protocols to approach challenging conversations, but the reality is that no two clinical conversations are the same. A successful approach in one setting may be inadequate or even problematic with another patient or family. Relational proficiency in medical interviews necessitates recognition of, and flexible response to, variable patient and family cues [34]. In the ASP context, this relational flexibility seems to be higher and learners have better suited communication skills for gathering information.

However, an interesting observation is that no difference was observed between the SRP and ASP groups for the item ‘Gathering information exploration of the patient’s problems to discover the patient’s perspective’. This item requires a more empathic approach for gathering information. In order to respond to this item, raters paid attention if the learner explored patient’s ideas, patient’s concerns regarding each problem, patient’s expectations, how each problem affects the patient’s life and if he/she encouraged the patient to express feelings. This result is in accordance with the fact that no differences were observed between the SRP and ASP groups concerning the ‘Building relationship’ CCG main task or empathy, as evaluated with the CARE score. During the SRP training, the learner who played the doctor role had not also played the role of the patient. In order to make the access to an empathic approach easier for the peer role play-group, students have to switch roles (doctor and patient), but it was not the case for the SRP training in this study [18, 35]. HM Bosse et al showed that having role-played both the doctor and patient in the exercise is a key to the success of peer role play and creates a heightened awareness for the ambiguity of roles of the partners in communication, facilitating a more empathic approach [18]. The ability to sense and appreciate patients’ views, as well as the empathic approach towards the patients represent the basis for a functioning and safe patient–physician relationship [36]. It improves patient adherence and supports patient safety which are increasingly important issues in physicians’ daily work [37, 38]. Most certainly, switching roles in peer role play should be mandatory during our communication skills training program in order to allow learners to experience both biomedical and patient perspectives.

With respect to non-verbal behaviour items, nervousness was significantly higher in the ASP group compared to the SRP group. The most frequent signs of nervousness observed by the raters were self-touching (rubbing one’s face, grooming the hair), frequent postural shift, manipulation of objects (mostly the pen) and inappropriate facial display such as smiling or laughing. The deeper authenticity of the ASP situation and the emotional commitment of the professional actors pushed learners at more important levels of emotion depth, which could explain the higher nervousness [19]. Self-touching or unpurposive movements are signs of anxiety, tension and preoccupation and these non-verbal behaviors are known to accompany a lower patient satisfaction [8, 39]. Repeating the ASP exercise could diminish nervousness, increase auto-control and improve learner’s performance. Concerning the inappropriate facial display, namely smiling or laughing at inappropriate moments during the medical interview, in the European culture, it appears to cover embarrassment [40]. Embarrassment is a feeling of concern with one’s public image and with the reactions from others to inappropriate behaviour [40]. Therefore learners in the ASP group present more embarrassment signals compared to the SRP group, as a sign of failure to present a desired image to the actors, whom they seem to regard as evaluators of their performance. The embarrassment signals have a disruptive effect upon social interaction therefore, repeating the ASP exercise could also diminish these signals and improve students’ performances.

Concerning the gender specific differences in CCG scores, female students performed significantly better for the task ‘Building the relationship - Using appropriate non-verbal behavior’ and ‘Initiating the session – Identifying the reasons for the consultation’. Our results are consistent with several studies showing that female physicians interact differently with the patients, compared to their male counterpart by showing more empathy, using more positive statements and more affective behavior when communicating diagnosis-specific information [41–46].

Several limitations to our study should be noted in interpreting these findings. First, its representativeness is weighed down by the small sample of students who participated in the study. However, the fact that all 20 students were evaluated independently by 3 raters increased the accuracy of the results. Second, the communication skills assessment was based on several different scenarios. It has been previously suggested that the feasibility of a communication skills assessment would depend on the difficulty of the contents of the medical interviews [47]. We consider that even though we used different scenarios, they were similarly challenging for the students. Third, the students from the ASP group were 1 year older than those from the SRP group. The more adequate skills of ASP group in terms of gathering information in order to discover the biomedical perspective, background information and context, but also in terms of closing the session with forward planning could also be due to their supplementary year of medical education. Forth, although non-verbal behaviors, like nervousness, are under less conscious control than verbal behaviors, and are generally irrepressible, students may have acted differently than in real-life situations because of the simulated nature of the consultation and because they were also being filmed and observed by their colleagues [8, 21]. However, the SRP and ASP workshops took place under the same conditions, therefore, if non-verbal behavior results were biased by these conditions, they were equally biased in both groups. Finally, the impact of the non-verbal behavior was assessed through an overall rating of posture and nervousness rather than a measure of specific behaviors.

## Conclusion

In this study, data presented suggested that the two communication skills training methods SRP and ASP are highly comparable for undergraduate medical students. However, it also suggested that the ASP method, pushes the learner to use more adapted communication skills for gathering biomedical and context information as well as for closing the session with forward planning. Our results did not show any educational benefit for the ASP method, regarding the empathic approach and students’ understanding of the patient’s perspective. Switching doctor-role with patient-role in the SRP setting might improve these skills in medical students. As the ASP approach generated significantly more frequent signs of nervousness, a new training program for the fifth year medical students’ had been introduced in the Faculty of Medicine of Brest curricula. This new training is based on drama exercises designed to heighten an awareness of sight, hearing, touch and proxemics in non-verbal communication.

## Supplementary Information

**Additional file 1: Supplementary Table 1.** Calgary-Cambridge Referenced Observation Guide adapted from Kurtz SM, 1998 and Silverman JD, 1998 [30, 31]

**Additional file 2: Supplementary Table 2.** CARE (Consultation and Relational Empathy) questionnaire adapted from Mercer SW, 2005 [26].

## Data Availability

Data may be available by request submitted to the corresponding author.

## Abbreviations

SRP: Student role play
ASP: Actor simulated patient
CCG: Calgary-Cambridge Referenced Observation Guide
CARE: Consultation and Relational Empathy

## References

[1] Buller MK, Buller DB (1987). Physicians' communication style and patient satisfaction. J Health Soc Behav.

[2] Kiesler DJ, Auerbach SM (2003). Integrating measurement of control and affiliation in studies of physician-patient interaction: the interpersonal circumplex. Soc Sci Med.

[3] Maguire P, Pitceathly C (2002). Key communication skills and how to acquire them. BMJ.

[4] Bertakis KD, Azari R (2011). Patient-centered care is associated with decreased health care utilization. J Am Board Fam Med.

[5] Stepien KA, Baernstein A (2006). Educating for empathy. A review. J Gen Intern Med.

[6] Vogel D, Meyer M, Harendza S (2018). Verbal and non-verbal communication skills including empathy during history taking of undergraduate medical students. BMC Med Educ.

[7] Mehrabian A, Ferris SR (1967). Inference of attitudes from nonverbal communication in two channels. J Consult Psychol.

[8] Ishikawa H, Hashimoto H, Kinoshita M, Fujimori S, Shimizu T, Yano E (2006). Evaluating medical students' non-verbal communication during the objective structured clinical examination. Med Educ.

[9] Di Blasi Z, Harkness E, Ernst E, Georgiou A, Kleijnen J (2001). Influence of context effects on health outcomes: a systematic review. Lancet.

[10] Mercer SW, Reynolds WJ (2002). Empathy and quality of care. Br J Gen Pract.

[11] Bikker AP, Mercer SW, Reilly D (2005). A pilot prospective study on the consultation and relational empathy, patient enablement, and health changes over 12 months in patients going to the Glasgow homoeopathic hospital. J Altern Complement Med.

[12] Silverman JD, Draper J, Kurtz SM (1995). The inhumanity of medicine. Interpersonal and communication skills can be taught. BMJ.

[13] Kurtz SM, Silverman JD (1996). The Calgary-Cambridge referenced observation guides: an aid to defining the curriculum and organizing the teaching in communication training programmes. Med Educ.

[14] Taylor S, Bobba S, Roome S, Ahmadzai M, Tran D, Vickers D, Bhatti M, De Silva D, Dunstan L, Falconer R (2018). Simulated patient and role play methodologies for communication skills training in an undergraduate medical program: randomized, crossover trial. Educ Health (Abingdon).

[15] von Fragstein M, Silverman J, Cushing A, Quilligan S, Salisbury H, Wiskin C (2008). Education UKCfCCSTiUM: UK consensus statement on the content of communication curricula in undergraduate medical education. Med Educ.

[16] May W, Park JH, Lee JP (2009). A ten-year review of the literature on the use of standardized patients in teaching and learning: 1996-2005. Med Teach.

[17] Bosse HM, Nickel M, Huwendiek S, Schultz JH, Nikendei C (2015). Cost-effectiveness of peer role play and standardized patients in undergraduate communication training. BMC Med Educ.

[18] Bosse HM, Schultz JH, Nickel M, Lutz T, Moltner A, Junger J, Huwendiek S, Nikendei C (2012). The effect of using standardized patients or peer role play on ratings of undergraduate communication training: a randomized controlled trial. Patient Educ Couns.

[19] Bell SK, Pascucci R, Fancy K, Coleman K, Zurakowski D, Meyer EC (2014). The educational value of improvisational actors to teach communication and relational skills: perspectives of interprofessional learners, faculty, and actors. Patient Educ Couns.

[20] Lane C, Rollnick S (2007). The use of simulated patients and role-play in communication skills training: a review of the literature to august 2005. Patient Educ Couns.

[21] DePaulo BM (1992). Nonverbal behavior and self-presentation. Psychol Bull.

[22] Ratka A (2018). Empathy and the development of affective skills. Am J Pharm Educ.

[23] Atkins D, Uskul AK, Cooper NR (2016). Culture shapes empathic responses to physical and social pain. Emotion.

[24] Simmenroth-Nayda A, Heinemann S, Nolte C, Fischer T, Himmel W (2014). Psychometric properties of the Calgary Cambridge guides to assess communication skills of undergraduate medical students. Int J Med Educ.

[25] Kurtz SM, Silverman J, Draper J (2005). Teaching and learning communication skills in medicine: Radcliffe pub (Oxford).

[26] Mercer SW, McConnachie A, Maxwell M, Heaney D, Watt GC (2005). Relevance and practical use of the consultation and relational empathy (CARE) measure in general practice. Fam Pract.

[27] Roberts BW, Trzeciak CJ, Puri NK, Mazzarelli AJ, Trzeciak S (2020). Racial and socioeconomic disparities in patient experience of clinician empathy: a protocol for systematic review and meta-analysis. BMJ Open.

[28] Wu H, Zhang Y, Li S, Liu Q, Yang N (2020). Care is the Doctor's best prescription: the impact of doctor-patient empathy on the physical and mental health of asthmatic patients in China. Psychol Res Behav Manag.

[29] Crosta Ahlforn K, Bojner Horwitz E, Osika W (2017). A Swedish version of the consultation and relational empathy (CARE) measure. Scand J Prim Health Care.

[30] Kurtz SMSJ, Draper J (1998). *Teaching and Learning Communication Skills in Medicine*: Radcliffe medical press (Oxford).

[31] Silverman JDKS, Draper J (1998). *Skills for Communicating with Patients*: Radcliffe medical press (Oxford).

[32] Landis JR, Koch GG (1977). An application of hierarchical kappa-type statistics in the assessment of majority agreement among multiple observers. Biometrics.

[33] Graf J, Smolka R, Simoes E, Zipfel S, Junne F, Holderried F, Wosnik A, Doherty AM, Menzel K, Herrmann-Werner A (2017). Communication skills of medical students during the OSCE: gender-specific differences in a longitudinal trend study. BMC Med Educ.

[34] Lingemann K, Campbell T, Lingemann C, Holzer H, Breckwoldt J (2012). The simulated patient's view on teaching: results from a think aloud study. Acad Med.

[35] Lim EC, Oh VM, Seet RC (2008). Overcoming preconceptions and perceived barriers to medical communication using a 'dual role-play' training course. Intern Med J.

[36] de Haes H, Bensing J (2009). Endpoints in medical communication research, proposing a framework of functions and outcomes. Patient Educ Couns.

[37] Levinson W, Roter DL, Mullooly JP, Dull VT, Frankel RM (1997). Physician-patient communication. The relationship with malpractice claims among primary care physicians and surgeons. JAMA.

[38] Street RL, Makoul G, Arora NK, Epstein RM (2009). How does communication heal? Pathways linking clinician-patient communication to health outcomes. Patient Educ Couns.

[39] RR HJA. Non-verbal aspects of empathy and rapport in physician–patient interaction. In: Non-verbal Communication in the Clinical Context: Press PSU; 1986. p. 36–73.

[40] Robert J, Edelmann JA, Contarello A, Georgas J, Villanueva C, Zammuner V (1987). Self-reported verbal and nonverbal strategies for coping with embarrassment and observer’s perceived reactions in five European cultures. Soc Sci Inf.

[41] Roter DL, Hall JA, Aoki Y (2002). Physician gender effects in medical communication: a meta-analytic review. JAMA.

[42] Roter DL, Hall JA (2004). Physician gender and patient-centered communication: a critical review of empirical research. Annu Rev Public Health.

[43] Swygert KA, Cuddy MM, van Zanten M, Haist SA, Jobe AC (2012). Gender differences in examinee performance on the step 2 clinical skills data gathering (DG) and patient note (PN) components. Adv Health Sci Educ Theory Pract.

[44] Bylund CL, Makoul G (2002). Empathic communication and gender in the physician-patient encounter. Patient Educ Couns.

[45] Dielissen P, Bottema B, Verdonk P, Lagro-Janssen T (2011). Attention to gender in communication skills assessment instruments in medical education: a review. Med Educ.

[46] Meeuwesen L, Bensing J, van den Brink-Muinen A (2002). Communicating fatigue in general practice and the role of gender. Patient Educ Couns.

[47] Hodges B, Turnbull J, Cohen R, Bienenstock A, Norman G (1996). Evaluating communication skills in the OSCE format: reliability and generalizability. Med Educ.

