# Supplementary material for: Simulated patient and role play methodologies for communication skills and empathy training of undergraduate medical students
Source: BMC Med Educ. 2020 Dec 4;20:491. doi: 10.1186/s12909-020-02401-0 (PMC7716460; doi:10.1186/s12909-020-02401-0)
Supplement: Supplementary file 1 — Additional file 1: Supplementary Table 1. Calgary-Cambridge Referenced Observation Guide adapted from Kurtz SM, 1998 and Silverman JD, 1998 [30, 31] [file 12909_2020_2401_MOESM1_ESM.docx]

**Supplementary Table 1.** Calgary-Cambridge Referenced Observation Guide adapted from Kurtz SM, 1998 and Silverman JD, 1998 [[46](#_ENREF_46), [47](#_ENREF_47)]

| CCG main tasks and descriptors | Not done | Incompletely done | Done |
| --- | --- | --- | --- |
| Initiating the session |  |  |  |
| Preparing the session |  |  |  |
| Establishing initial rapport |  |  |  |
| Identifying the reasons for the consultation |  |  |  |
| Gathering information - exploration of the patient’s problems to discover the: |  |  |  |
| biomedical perspective |  |  |  |
| the patient’s perspective |  |  |  |
| background information - context |  |  |  |
| Explanation and planning |  |  |  |
| Providing the correct amount and type of information |  |  |  |
| Aiding accurate recall and understanding |  |  |  |
| Achievieng a shared understanding : incorporating the patient’s illness framework |  |  |  |
| Planning : shared decision making |  |  |  |
| Building the relationship |  |  |  |
| Using appropriate non-verbal behaviour |  |  |  |
| Developping rapport |  |  |  |
| Involving the patient |  |  |  |
| Providing structure |  |  |  |
| Making organisation overt |  |  |  |
| Attending to flow |  |  |  |
| Closing the session |  |  |  |
| Forward planning |  |  |  |
| Ensuring appropriate point of closure |  |  |  |
