# Supplementary material for: Simulated patient and role play methodologies for communication skills and empathy training of undergraduate medical students
Source: BMC Med Educ. 2020 Dec 4;20:491. doi: 10.1186/s12909-020-02401-0 (PMC7716460; doi:10.1186/s12909-020-02401-0)
Supplement: Supplementary file 2 — Additional file 2: Supplementary Table 2. CARE (Consultation and Relational Empathy) questionnaire adapted from Mercer SW, 2005 [26]. [file 12909_2020_2401_MOESM2_ESM.docx]

**Supplementary Table 2.** CARE (Consultation and Relational Empathy) questionnaire adapted from Mercer SW, 2005 [[26](#_ENREF_26)]

| **How good was the practitioner at...** | | **Poor** | **Fair** | **Good** | **Very good** | **Excellent** |
| --- | --- | --- | --- | --- | --- | --- |
| 1) Making you feel at ease | (introducing him/herself, explaining his/her position, being friendly and warm towards you, treating you with respect; not cold or abrupt) |  |  |  |  |  |
| 2) Letting you tell your "story" | (giving you time to fully describe your condition in your own words; not interrupting, rushing or diverting you) |  |  |  |  |  |
| 3) Really listening | (paying close attention to what you were saying; not looking at the notes or computer as you were talking) |  |  |  |  |  |
| 4) Being interested in you as a whole person | (asking/knowing relevant details about your life, your situation; not treating you as "just a number") |  |  |  |  |  |
| 5) Fully understanding your concerns | (communicating that he/she had accurately understood your concerns and anxieties; not overlooking or dismissing anything ) |  |  |  |  |  |
| 6) Showing care and compassion | (seeming genuinely concerned, connecting with you on a human level; not being indifferent or "detached") |  |  |  |  |  |
| 7) Being positive | (having a positive approach and a positive attitude; being honest but not negative about your problems) |  |  |  |  |  |
| 8) Explaining things clearly | (fully answering your questions; explaining clearly, giving you adequate information; not being vague) |  |  |  |  |  |
| 9) Helping you to take control | (exploring with you what you can do to improve you health yourself; encouraging rather than "lecturing" you) |  |  |  |  |  |
| 10) Making a plan of action with you | (discussing the options, involving you in decisions as much as you want to be involved; not ignoring your views) |  |  |  |  |  |
